# Influence of mandibular and palatal intraoral appliances on erosion *in situ* study outcome

**DOI:** 10.1590/1678-7757-2018-0153

**Published:** 2019-01-14

**Authors:** Maisa Camillo Jordão, Franciny Querobim Ionta, Bianca Tozi Portaluppe Bergantin, Fernanda Lyrio Mendonça, Natália Mello dos Santos, Heitor Marques Honório, Thais Marchini de Oliveira, Daniela Rios

**Affiliations:** * Universidade de São Paulo, Faculdade de Odontologia de Bauru, Departamento de Odontopediatria, Ortodontia e Saúde Coletiva, Bauru, São Paulo, Brasil

**Keywords:** Dental erosion, Enamel, Protocols

## Abstract

The standardization of *in situ* protocols for dental erosion is important to enable comparison between studies.

**Objective:**

Thus, the objectives of this study were to evaluate the influence of the location of *in situ* intraoral appliance (mandibular X palatal) on the extent of enamel loss induced by erosive challenges and to evaluate the comfort of the appliances.

**Material and Methods:**

One hundred and sixty bovine enamel blocks were selected according to their initial surface hardness and randomly divided into two groups: GI - palatal appliance and GII - mandibular appliance. Twenty volunteers wore simultaneously one palatal appliance (containing 4 enamel blocks) and two mandibular appliances (each one containing 2 enamel blocks). Four times *per* day during 5 days, the volunteers immersed their appliances in 0.01 M hydrochloric acid for 2 minutes, washed and reinserted them into the oral cavity for 2 hours until the next erosive challenge. After the end of the *in situ* phase, the volunteers answered a questionnaire regarding the comfort of the appliances. The loss of tissue in the enamel blocks was determined profilometrically. Data were statistically analyzed by paired t-test, Chi-square and Fisher's Exact Test (p<0.05).

**Results:**

The enamel blocks allocated in palatal appliances (GI) presented significantly higher erosive wear when compared to the blocks fixed in mandibular appliances (GII). The volunteers reported more comfort when using the palatal appliance.

**Conclusions:**

Therefore, the palatal appliance is more comfortable and resulted in higher enamel loss compared to the mandibular one.

## Introduction

For many years, erosive tooth wear received little attention by dental professionals and researchers.^1^ However, the high prevalence of dental erosion has changed this scenario.^1^ ^,^ ^2^ Dental erosion has become a daily concern in clinical dental practice and anti-erosive agents have been increasingly investigated within the last decades.^1^

Randomized clinical trials offer the highest level of scientific evidence; however, it is very difficult to obtain precise clinical measures of erosive tissue loss.^3^ Alternatively, *in situ* studies can be conducted to overcome methodological difficulties faced by *in vivo* studies. *in situ* studies have many advantages, such as reduced number of volunteers, shorter time required and possibility to control the acid challenge.^3^ The main advantage of *in situ* models of dental erosion is the exposure of specimens to saliva.^3^ It is known that saliva provides protection against dental erosion^4^ ^–^ ^6^ and can dilute, neutralize, and buffer acids in the oral cavity.^6^ Also, saliva can provide calcium, phosphate and fluoride to dental enamel^6^ and it plays an important role in the formation of the acquired enamel pellicle, which diminishes the contact between acids and enamel.^4^ ^,^ ^5^

Saliva can present qualitative and quantitative differences depending on the gland secreting it.^7^ The parotid glands secrete saliva rich in amylase and proline-rich proteins, while saliva from sublingual and submandibular glands contains high concentration of lysozyme and mucin.^4^ Proteins of the acquired enamel pellicle change according to the location in the dental arches, which might impact their ability to protect against erosion.^8^ In addition, the site of erosive lesions appear to correlate with a thin dental pellicle.^9^ On the other hand, buffering capacity and flow rate are decreased in sites bathed by mucous saliva.^10^ Clinically, it is known that the palatal surface of upper incisors is more likely to develop erosion than the lingual surface of lower teeth.^6^ However, recent studies using intraoral appliances to assess dental erosion have shown that the location of the appliance do not interfere in the rehardening effect of saliva on eroded enamel^11^ and in the protective effect of saliva against initial erosive demineralization.^12^ Nonetheless, these studies did not consider the whole process of successive erosive cycles of demineralization and rehardening.

Valuable data regarding preventive measures for dental erosion have been obtained from *in situ* studies.^3^ However, the location of the intraoral appliance differs among different research groups^13^ and whether this can influence the degree of enamel loss or the effect of the studied preventive measure is not known. Thus, the first step is to investigate the extent in which the type of oral device may interfere on the enamel loss in *in situ* erosive cycles. To diminish confounding factors, the appliances should be tested for erosion alone, without any treatment.

Another important point is the volunteer collaboration and comfort while using the intraoral appliance, which can influence the results of the experiment. However, no information related to the volunteer's comfort during the use of mandibular or maxillary oral appliances is currently available.

Therefore, the aim of this study was to investigate the influence of the location (mandibular × palatal) of intraoral appliances on the degree of enamel loss caused by erosive challenges. The volunteers' report on the comfort of the appliances was also evaluated.

## Materials and methods

### Experimental design

**Figure 1:**
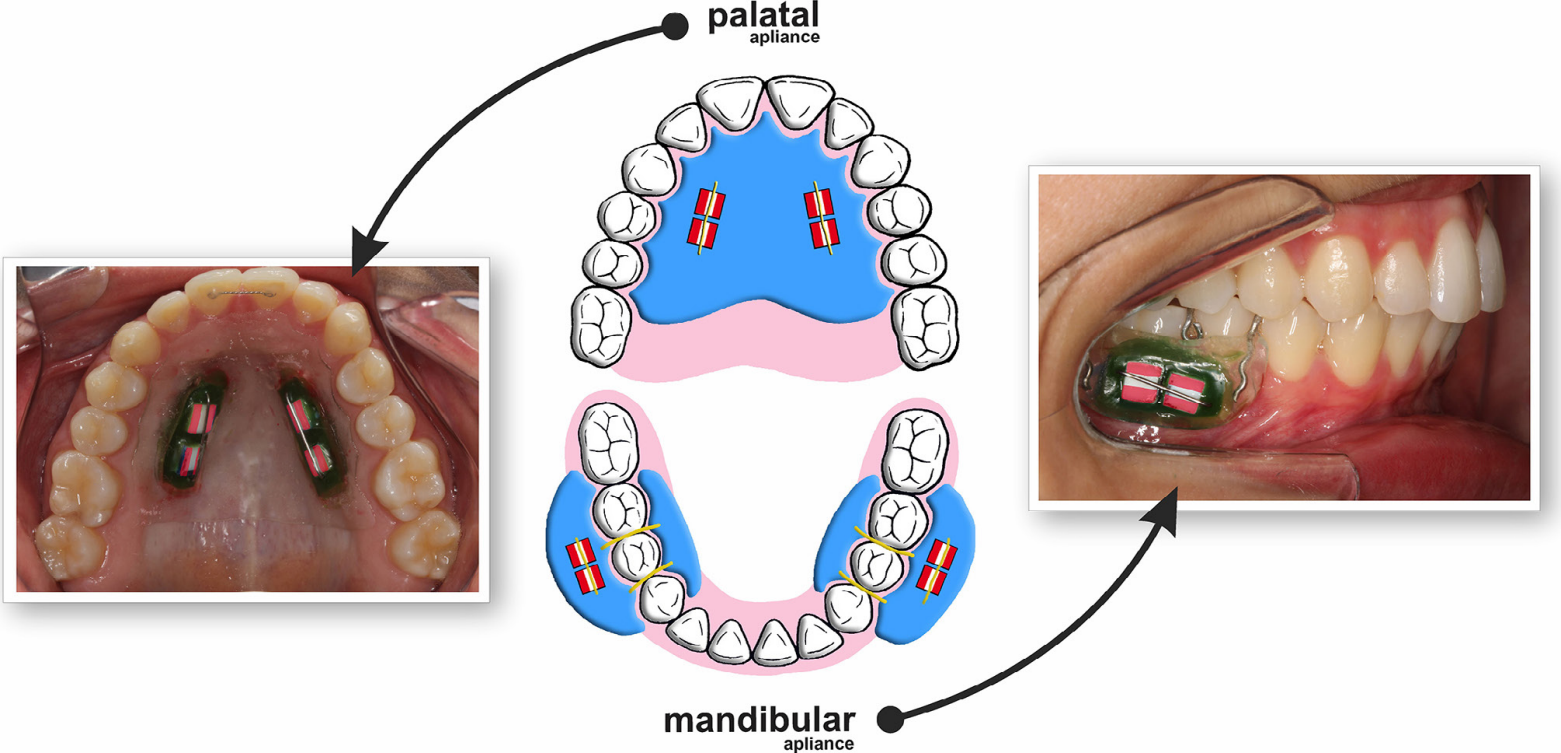
Characteristics of the palatal and mandibular appliances

This study was conducted under a single-blind randomized *in situ* design. Bovine enamel blocks (n=160) were selected by initial surface hardness and randomly divided into two groups: GI - palatal appliance and GII - mandibular appliance. Each volunteer (n=20) wore at the same time one acrylic palatal appliance (containing 4 enamel blocks) and two acrylic mandibular appliances (each containing 2 enamel blocks) (Figure 1). The comfort of using the appliances was evaluated by a questionnaire. The erosive cycle procedure consisted on immersing the appliances in 0.01 M hydrochloric acid pH 2.3 for 2 minutes 4 times *per* day for 5 days. The response variable was tissue loss determined profilometrically.

### Enamel block preparation

Two hundred enamel blocks (4×4×3 mm) were prepared from extracted bovine incisors. The blocks were cut using a cutting machine (Isomet Low Speed Saw, Buehler Ltd.; Lake Bluff, Illinois, United States) and two diamond disks (Extec Corp.; Enfield, Connecticut, United States) separated by a 4-mm thick spacer. The blocks' surfaces were ground flat with water-cooled silicon carbide discs (320, 600, and 1200 grades of Al_2_O_3_ papers; Extec Corp.; Enfield, Connecticut, United States) and polished with felt paper wet by diamond spray (1 μm; Buehler Ltd.; Lake Bluff, Illinois, United States). The enamel blocks were cleaned in ultrasonic bath with deionized water for 10 min between the polishing steps. Surface hardness was determined by performing five indentations at 100-μm distance from each other on the center of each block (Knoop diamond, 25 g, 10 s, Hardness tester from Buehler, Lake Bluff, Illinois, United States). One hundred and sixty enamel blocks with mean hardness of 350 (±14) KPa/mm^2^ were selected and randomly allocated to volunteers and appliances using Excel software.

### Initial profilometry

The buccal surfaces of the enamel blocks (4×4 mm) were marked with a scalpel blade (Embramac, Itapira, São Paulo, Brazil) to define a 1-mm control area (at the border) and 2-mm test area (at the center) in width. The initial profile of enamel blocks was evaluated by Marh's contact profilometer (MarSurf GD 25, Marh, Göttingen, Lower Saxony, Germany) coupled to a computer with a contour software (MarSurf XCR 20,Marh, Göttingen, Lower Saxony, Germany). Enamel blocks were fixed to a special holder to standardize their initial and final analysis position. Five readings were made in each block at the following distances of the relative position of the block on the y-axis: 2.25, 2.0, 1.75, 1.5, and 1.25 μm. Each profile reading was saved individually.

Before the *in situ* phase, the blocks were sterilized with ethylene oxide.^14^ The borders of enamel blocks were protected with cosmetic nail varnish (Maybelline Colorama: Cosbra Cosmetics Ltda, São Paulo, São Paulo, Brazil) and served as control areas (no acid exposure during the *in situ* phase) for profilometric tissue loss measurement.

### *In situ* phase

This study was approved by the local Research Ethics Committee (protocol number 24216514.8.0000.5417) and conducted in full accordance with the Declaration of Helsinki. Informed consent was obtained from each volunteer at the beginning of the study, prior to confirmation of their eligibility. Participants had the right to withdraw from the study at any time and for any reason without prejudice.

Twenty healthy adult volunteers (aged 18–29 years) participated in this study after satisfying the following inclusion criteria: residing in the same fluoridated area with 0.70 mg F/L, physiologically stimulated salivary flow rate >1 mL/min, adequate oral health with no caries, erosion lesions, or significant gingivitis/periodontitis. The exclusion criteria were systemic illness, pregnancy or breastfeeding, under orthodontic intervention, and professional application of fluoride compounds in the last two months.

The intraoral palatal and mandibular appliances were made with acrylic resin on a plaster model. The palatal appliance had two vertical rows, one on the right and the other on the left side, with one cavity (10×4×4 mm) for the fixation of two enamel blocks on each side (four blocks *per* appliance, Figure 1). The mandibular appliance had only one cavity on the buccal side for the fixation of two enamel blocks. Two mandibular appliances (to be used on the right and left sides, each side with two blocks) were confectioned for each volunteer. The mandibular appliances were made with acrylic resin and were fixed on the right and left first molars by Adams clasps^3^ ^,^ ^12^ (Figure 1). The enamel blocks were fixed with wax in the appliances. An orthodontic wire was attached to the ends of the cavity (passing over but without touching the enamel blocks) in order to prevent abrasion of the blocks by tongue and soft tissue. The position of the enamel blocks was randomly determined for each volunteer and each appliance.

Seven days prior to and during all the experimental phase, the volunteers brushed their teeth with a standardized toothbrush (Curaprox 5460 ultra-soft: Curaden AG, Kriens, Switzerland) and fluoride toothpaste (Tripla Ação® Colgate: Palmolive Comercial Ltda., São Paulo, São Paulo, Brazil). They were instructed to brush their teeth after meals without the appliances in their mouths and not to use any other fluoride product.

The volunteers received written instructions and they were properly trained prior to the experimental *in situ* phase. The appliances were worn during sleep on the night prior to the beginning of the experiment to allow the formation of the acquired pellicle. Thereafter, the upper and lower appliances were simultaneously used for 5 days from 7 am to 6 pm, being removed during meals (for 1 h 45 min)^10^ ^,^ ^16^ ^–^ ^17^. When out of the oral cavity, the appliances were stored in a plastic box wrapped in gauze wet with tap water (Bauru, São Paulo, Brazil - 0.7 ppm F) to prevent dehydration of the enamel. Tooth erosion was simulated by extraoral immersion of the appliances into 150 mL of 0.01 M hydrochloric acid, pH 2.3, at room temperature for 2 min. This procedure was performed *ex-vivo* to protect teeth from potential damage. Then, the volunteers washed the appliances with tap water and put them on until the next challenge.^18^ The experimental protocol consisted of: 7 am - appliance worn for pellicle rehydration; 8.00 am - erosive challenge; 10.00 am - erosive challenge; 12.00 am - lunch time (stored in wet gauze); 1.45 pm - appliance worn for pellicle rehydration; 2.00 pm - erosive challenge; 4.00 pm - erosive challenge; 6.00 pm - appliance removal.

### Final profilometry

After the *in situ* phase, the enamel blocks were removed from the intraoral appliances. The cosmetic nail varnish was carefully removed from the surface by means of mechanical displacement from the enamel border. Enamel blocks were repositioned on the special holder on the profilometer table according to its initial position. Five readings were performed using the same software (MarSurf XCR 20, Marh, Göttingen, Lower Saxony, Germany) and measurement parameters described above (initial profilometry).

**Figure 2:**
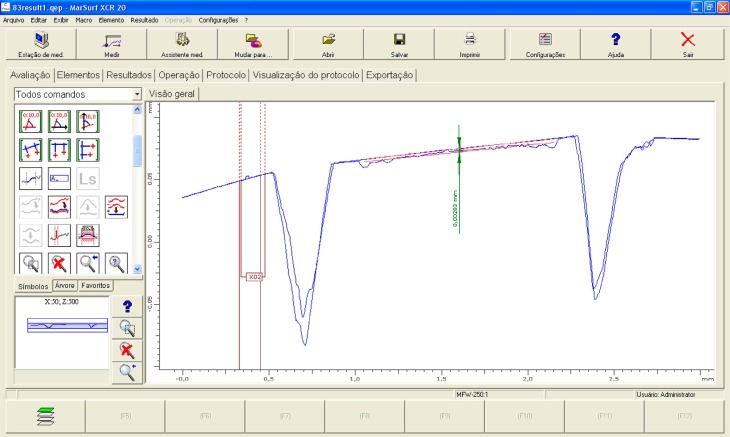
Superimposition of initial and final profiles and measurement of enamel loss

For each of the five graphs, initial and final profiles were superimposed using the application XCR 20 (Marh, Göttingen, Lower Saxony, Germany). Parallel regression lines were constructed with a length of 0.5 mm on each initial and final profile. The vertical distance between the regression lines was defined as the amount of tissue loss (μm) (Figure 2). The enamel loss of each block was reported as the mean of five graphs.

### Comfort evaluation

At the end of the *in situ* phase, the volunteers received a questionnaire regarding the comfort of the appliances during use and speech, and sensitivity during use or after appliance removal, with yes or no as possible answers (dichotomized questions). As last question, the volunteers were asked which appliance location they preferred (palatal or mandibular) given the possibility of volunteering in future studies.

### Statistical analysis

Statistical analysis was performed with SigmaPlot version 12.3 (Systat Software GmbH; Erkrath, North Rhine-Westphalia, Germany). The assumptions of equality of variances and normal distribution of errors were checked for erosive enamel loss. Since the assumptions were met, the paired t-test was applied. Chi-square or Fisher Exact Test were applied for the association analysis between appliance location and comfort questions. The level of significance was set at 5%.

## Results

All volunteers completed the *in situ* study and followed the protocol. Table 1 shows the mean enamel loss of each experimental group. The enamel blocks of palatal appliances (GI) presented significantly higher enamel loss compared to enamel blocks on mandibular appliances (GII).

**Table 1:** Means and standard deviation of enamel loss (μm) for the mandibular and palatal appliances

Experimental Groups	Enamel loss (±sd)
GI (Palatal Appliance)	1.91 (± 0.95)ᵃ μm
GII (Mandibular Appliances)	1.36 (± 0.65)^b^ μm

* Groups whose means are followed by distinct letters differ significantly (Paired t-test, p=0.018)

Table 2 shows the results on appliance comfort from 18 volunteers. The mandibular appliance was associated with discomfort during speech (p=0.003), discomfort during use for 5 days (p=0.001), sensitivity during use (p=0.008), and sensitivity after appliance removal (p=0.001). All volunteers preferred the palatal appliance in potential future studies.

**Table 2:** Percent of volunteers (n=18) with discomfort and sensitivity for the palatal and mandibular appliances

Experimental Groups	GI (Palatal Appliance)	GII(Mandibular Appliances)
Discomfort on speech	16.6%	72.2%
Discomfort on use for 5 days	11.1%	72.2%
Pain on use	0%	39.9%
Pain after appliance removal	0%	50%
Number of days with pain after appliance removal	-	1 to 2 days

## Discussion

The results showed that the intraoral location of enamel blocks subjected to erosive challenge in *in situ* studies could affect enamel loss. The blocks in palatal appliances in the upper jaw presented higher enamel loss compared to the ones in buccal appliances in the lower jaw. Although small, the difference was significant and in line with epidemiological studies on erosion sites, which show that palatal surfaces of maxillary incisors and occlusal surfaces of mandibular molars are the areas most affected by erosion.^19^ ^–^ ^22^ The effect of oral site on the degree of tooth erosion might be explained by variations on the flow and composition of saliva^15^ from different salivary glands, which are located in different sites of oral cavity.^4^ Faster pH recovery after ingestion of orange juice was observed on the second mandibular premolar compared to the maxillary central incisor due to the proximity of the tooth to the parotid gland.^16^ During stimulation, parotid glands are the major contributors to the salivary flow rate, and their main role is related to the buffer capacity by the increase of bicarbonate concentration.^6^ ^,^ ^10^ In this study, the exposure of enamel blocks to acid did not fully simulate a clinical situation, since it was performed extra-orally and the blocks were washed before appliance reinsertion, impairing the clearance and buffering effect of saliva.^6^ ^,^ ^15^ On the other hand, the presence of the appliances in the mouth is itself a mechanical stimulus for salivary flow. The stimulated salivary flow rate promotes an increase in calcium and phosphate, which could benefit eroded enamel rehardening.^11^ ^,^ ^18^ ^,^ ^23^ The blocks located on the buccal site in the mandibular appliances, which are closer to the parotid glands than the palatal ones, might have had a higher degree of enamel rehardening. However, it has been proposed that the rehardening of erosive lesions is not a true remineralization because the partly dissolved crystal does not regrow;^24^ rather, a deposition of amorphous mineral occurs on top of the eroded enamel prisms.^25^ In addition, whether the rehardened enamel is less susceptible to subsequent enamel loss by erosive challenge is not known. Therefore, we hypothesize that the results observed in this study had little influence of the rehardening effect of saliva.

Flow rate increase is not the only salivary mechanism to counteract the erosive challenge. Saliva, together with the gingival crevicular fluid and oral mucosa, are responsible for the formation of a bacteria-free organic layer by selective adsorption of proteins on the enamel surface, known as acquired enamel pellicle (AEP).^5^ ^,^ ^13^ ^,^ ^26^ AEP acts as a semi-permeable barrier between the tooth surface and the oral cavity, modulating the mineralization/demineralization processes.^5^ ^,^ ^26^ One study found that the AEPs formed near the duct orifices of the parotid and submandibular/sublingual salivary glands do not differ regarding protection of enamel against 0.1% and 1% citric acid attack of 30 and 60 s.^4^ However, when exposing pellicle-covered enamel blocks to 1% citric acid for 5 min, the AEP on the buccal aspect of the upper molars was less effective in protecting the enamel against demineralization compared to the AEP on the lingual aspect of the lower incisors.^4^ The authors suggested that specific components of the AEP at the lingual site such as mucin might be more effective after several minutes.^4^ The results of the present study are in line with the above-mentioned study.^4^ The acid challenge was performed with 0.01 M hydrochloric acid for 40 minutes (2 min 4x *per* day for 5 days) and a higher erosion was observed on blocks of maxillary palatal appliances compared to the blocks of mandibular buccal appliances. The pellicles formed at the buccal aspect of the lower molars are influenced by the parotid and submandibular/sublingual salivary glands, whereas the palatal aspect of the upper incisors is bathed by minor mucous glands. In contrast, when enamel blocks were previously exposed to saliva by palatal or mandibular intraoral appliances and then subjected to short-time acid exposure (0.01 M hydrochloric acid for 30 s), no difference was observed in enamel hardness.^12^ The previous and present studies reinforce the hypothesis that differences between AEP formed in palatal and mandibular buccal areas may be seen only after several minutes of acid challenge.^4^

Differences in enamel loss due to the location of the intraoral appliance might also reflect the AEP thickness, which varies within the dental arch and tooth surface. The AEP is thinner in the palatal surface of anterior maxillary teeth and thicker on the lingual surface of the lower posterior teeth.^9^ In this study, the AEP composition and ultrastructure were not assessed. Mucin, an important component of saliva and AEP, is not present in parotid saliva, being synthesized by minor mucous glands and by submandibular and sublingual glands.^10^ Mucins act as an important lubricant, therefore, sites in the oral cavity bathed by saliva from submandibular and sublingual glands show more resistance to abrasion from soft tissues and tongue.^9^ ^,^ ^10^ The lubrication effect of mucin did not play a role in the present results because protective wires were used over the enamel blocks. This procedure was included in the experimental design since it is generally present in intraoral appliances of previous *in situ* studies^11^ ^,^ ^27^ to avoid the incidence of mechanical forces. A previous study showed that tongue abrasion enhances loss of eroded enamel.^28^ However, in the present study, the higher enamel loss seen in the blocks of the maxillary appliances might not be a consequence of tongue abrasiveness, since the wire inhibited the contact between enamel and tongue. In addition, the lack of mechanical impact must have reduced the disruption of the partially demineralized eroded enamel, which resulted in low values of enamel wear, despite the severity of the erosive challenge.

One of the difficulties of *in situ* studies is protocol compliance by volunteers.^29^ ^,^ ^30^ The intraoral appliance with enamel blocks has to be comfortable in order to increase volunteer collaboration. Both appliances of this study were designed based on volunteers' safety and comfort. However, all participants preferred the maxillary appliance, reporting that for the palatal appliance, the speech difficulty was related to the restriction of tongue movements and for the mandibular appliance, to cheek movements. They also described that the use of the mandibular appliance caused more speech difficulty. This result was unexpected, since the palate has an important role on pronunciation. It is hypothesized that the simultaneous use of the maxillary and mandibular appliances interfered on speech and the volunteers complained of the mandibular one because it was more uncomfortable to use. The mandibular appliance design was chosen based on a previously description of an intermittent mandibular appliance model for tooth erosion.^3^ ^,^ ^30^ The Adams clasp – used to hold the mandibular appliances to the molars – together with the pressure of the acrylic on the alveolar ridge might have been the reason for the sensitivity described by the volunteers. However, in a previous study that used another design for the mandibular appliance, similar to a soft silicon mouth guard, the volunteers also reported discomfort and occlusion interference.^11^ Thus, further studies are required to investigate a more comfortable design for mandibular appliances.

The effectiveness of the preventive measures depends on the severity of the erosive challenge. For example, the effect of fluoride appears to be reduced in a more severe acid attack.^31^ ^,^ ^32^ Therefore, knowing the degree of enamel loss for each study protocol is important. Our results show that palatal appliances might mimic more severe erosive challenges than mandibular appliances when using the present study design (*in situ* with hydrochloric acid). However, the present appliances might not reflect the results of other types of appliances.

## Conclusion

The use of palatal appliances resulted in higher enamel loss than the mandibular one when enamel blocks were subjected to erosive cycling. In addition, volunteers preferred the palatal appliance in terms of comfort.
